# Cognitive insight in patients with depression and non-suicidal self-injury

**DOI:** 10.3389/fpsyg.2026.1748116

**Published:** 2026-06-10

**Authors:** Chun Li, Chen Chen, Haozhe Li, Wen Li, Yuhua Zhu, Min Guo

**Affiliations:** 1 Shanghai Key Laboratory of Forensic Medicine, Key Laboratory of Forensic Science, Ministry of Justice, Shanghai Forensic Service Platform, Academy of Forensic Science, Shanghai, China; 2 Chenzhou Mental Hospital, Chenzhou, Hunan, China

**Keywords:** case-control study, cognitive insight, depression, healthy control, non-suicidal self-injury

## Abstract

**Background:**

Cognitive insight, a higher-order insight encompassing metacognitive processes, is directly linked to patients’ help-seeking behavior and disease self-management. Non-suicidal self-injury (NSSI) and depression are closely comorbid major global public health problems. Existing studies show depressed patients with NSSI have executive dysfunction, which may further impair metacognitive abilities. However, research on cognitive insight in this population remains scarce to date. This study aimed to investigate cognitive insight deficits in these patients, to provide evidence for precise clinical evaluation, individualized treatment, and risky behavior intervention.

**Methods:**

We enrolled 68 depressed patients with NSSI, 40 without NSSI, and 32 healthy controls. All completed the Beck Cognitive Insight Scale (BCIS); depressed patients also completed the Ottawa Self-Injury Inventory (OSI) and 24-item Hamilton Depression Rating Scale (HAMD-24). Participants were grouped by NSSI status, further divided into mild and severe NSSI subgroups, stratified by age into adolescent and adult subgroups. Data were analyzed via chi-square, *t*-test, ANOVA, Pearson correlation, and stepwise multiple linear regression.

**Results:**

In the total cohort, the severe NSSI subgroup showed lower self-reflectiveness and composite index, and higher total HAMD-24 score, cognitive disturbance and despair factors versus the mild subgroup (all *P* < 0.05). Composite index was negatively correlated with total HAMD-24 score (*r* = −0.366, *P* < 0.05). Regression revealed that cognitive disturbance factor independently predicted self-reflectiveness (β = −0.326, *P* < 0.05), and total HAMD-24 score predicted composite index (β = −0.377, *P* < 0.05). In adolescents, the severe NSSI subgroup showed lower self-reflectiveness and composite index scores, and higher total HAMD-24, cognitive disturbance, and diurnal variation scores versus the mild subgroup (all *P* < 0.05). BCIS were negatively correlated with total HAMD-24 score and diurnal variation (r = −0.514, −0.559, −0.505, −0.538; all *P* < 0.05). Regression showed total HAMD-24 score independently predicted self-reflectiveness (β = −0.734, *P* < 0.05), and diurnal variation factor predicted composite index (β = −0.583, *P* < 0.05). In adults, no significant between-group differences were found in BCIS (*P* > 0.05).

**Conclusion:**

This study demonstrates that patients with depression and severe NSSI show impaired cognitive insight, which correlates significantly with depression severity. Notably, this association shows a clear age-dependent pattern, present only in adolescent patients. For adolescents with depression and NSSI, targeted interventions for depressive symptoms, cognitive impairment, and circadian abnormalities may improve cognitive insight and reduce NSSI risk.

## 1 Introduction

Insight is a pivotal and complex construct in the clinical diagnosis and management of mental disorders. It is not only closely associated with patients’ help-seeking behavior and treatment adherence, but also carries profound practical implications for improving disease intervention efficacy, alleviating disease burden, reducing self-harm, suicidal and impulsive aggressive behaviors, and enhancing risk prevention and control capabilities. In clinical practice, a substantial proportion of patients with mental disorders present with impaired insight. Conceptually, insight can be dichotomized into clinical insight and cognitive insight. Clinical insight specifically refers to an individual’s ability to accurately appraise their pathological condition, manifested as voluntarily acknowledging the presence of a mental disorder, identifying abnormal mental states as pathological, and recognizing the necessity of standardized treatment (David, 1990). However, such verbalized illness awareness and treatment acceptance often merely reflect patients’ passive repetition of information from the surrounding clinical environment, failing to authentically reflect their capacity to integrate the disease-related information into their cognitive processes and effectively distinguish and renounce pathological false beliefs (Orfei et al., 2013). Cognitive insight is fundamentally distinct from clinical insight, defined as the ability to evaluate and rectify distorted beliefs and erroneous cognitions (Beck et al., 2004). This domain of insight does not involve awareness of mental illness *per se*, but centers on an individual’s awareness of their own thinking patterns and cognitive processes (Mervis et al., 2021). Specifically, it encompasses the ability to question personal judgments and cognitive biases, disengage from distorted cognitions, re-evaluate existing beliefs, and identify erroneous conclusions (Beck and Warman, 2004; Beck et al., 2004). Encompassing metacognitive processes (Konstantakopoulos, 2019), cognitive insight also reflects patients’ cognitive flexibility regarding their beliefs, judgments and subjective experiences, thereby representing a higher-order form of insight.

Non-suicidal self-injury (NSSI) not only inflicts physical and psychological harm on affected individuals, but also imposes a heavy burden on families, schools and society. Moreover, NSSI serves as a critical risk factor for suicidal behaviors (Chen H. et al., 2023). NSSI is formally defined as direct, intentional self-inflicted bodily harm in the absence of suicidal intent, which is not sanctioned by social or cultural norms; typical NSSI behaviors include intentional tissue damage or alteration, such as skin cutting, scratching, burning and biting. This clinical entity has been recognized as a novel diagnostic category in the Diagnostic and Statistical Manual of Mental Disorders, 5th Edition (DSM-5). Clinical studies have documented a high prevalence of NSSI among patients with mental disorders, and NSSI is closely linked to a range of psychiatric comorbidities (Briere and Gil, 1998), including major depressive disorder, substance use disorders and personality disorders (Nock et al., 2006). A meta-analysis has confirmed a strong association between NSSI and depression (Fox et al., 2015), and significant correlations between depressive symptoms and NSSI have been consistently reported in both community and clinical cohorts of adolescents and young adults (Poudel et al., 2022). Notably, patients with depression exhibit a significantly higher risk of NSSI than those without depression (Jacobson et al., 2015). A cross-sectional study of 12,068 adolescents across 11 European countries revealed that adolescents with depression had a 2.78-fold higher risk of NSSI compared with their non-depressed peers (Brunner et al., 2014).

Patients with depression represent a heterogeneous population, and not all such individuals engage in NSSI. Among depressed patients, comorbidity exacerbates disease severity and clinical complexity, and is associated with more severe social dysfunction, aggravated depressive symptoms, prolonged depressive episodes, and an increased frequency of suicide attempts (Auerbach et al., 2014). Existing research indicates that individuals with comorbid NSSI and depressive disorders carry the highest suicide risk (Knorr et al., 2016). Thus, the co-occurrence of NSSI and depression may exert unique impacts on the clinical treatment and prognosis of affected patients. In this context, targeted research on depressed patients with comorbid NSSI is of great significance for further clarifying the clinical characteristics of this population, developing tailored intervention strategies, improving patient prognosis, and reducing the overall disease burden.

To date, research focusing on cognitive insight in depressed patients with comorbid NSSI remains limited. A meta-analysis investigating insight in psychiatric patients identified that cognitive insight is associated with specific brain regions implicated in executive function (Pijnenborg et al., 2020). Executive function refers to the neurocognitive capacity that enables individuals to generate goal-directed thoughts, suppress irrelevant cognitions, and flexibly switch cognitive strategies as required (Mervis et al., 2021). Notably, executive function is closely intertwined with metacognition: existing evidence demonstrates that enhanced executive function predicts stronger metacognitive ability (Lundin et al., 2020), whereas executive function deficits may trigger impaired metacognition (Lysaker et al., 2008). Accumulating studies confirm that individuals engaging in NSSI present with executive dysfunction (Fikke et al., 2011), and research among depressed adolescents has also verified executive function impairment in those with comorbid NSSI (Zhang et al., 2022). Accordingly, we hypothesized that depressed patients with NSSI exhibit more severe cognitive insight deficits relative to those without NSSI. In the present study, the Beck Cognitive Insight Scale (BCIS) (Beck et al., 2004) was adopted to evaluate cognitive insight, and intergroup differences in cognitive insight and related indicators were compared across three cohorts: depressed patients with NSSI, depressed patients without NSSI, and healthy controls. Given that NSSI is highly prevalent in adolescents, with peak incidence at 15–17 years old and gradual attenuation after adulthood (Plener et al., 2015), age-stratified subgroup analyses were performed. This study aims to provide a scientific reference for clinical evaluation, personalized treatment development, and targeted prevention and intervention of risky behaviors in this high-risk patient population.

## 2 Materials and methods

### 2.1 Patients

This study was performed in strict compliance with the Declaration of Helsinki. Participants diagnosed with non-psychotic depressive disorder were recruited from inpatients at Chenzhou Mental Hospital between June 1, 2023, and May 30, 2025, and were divided into two subgroups: the depression with NSSI group (NSSI group) and the depression without NSSI group (non-NSSI group). The healthy control group (HC group) was composed of community-sourced volunteers, matched to the depression groups for gender, age and educational level. Written informed consent was obtained from all participants before study initiation; for participants under 18 years old, written informed consent was provided by both the participants and their legal guardians or close relatives. This study was approved by the Ethics Committee of Chenzhou Mental Hospital.

Inclusion Criteria for the NSSI Group: ➀ Met the diagnostic criteria for both depressive disorder and NSSI as defined in the Diagnostic and Statistical Manual of Mental Disorders, 5th Edition (DSM-5); ➁ Scored ≥ 1 on the past-year self-injury frequency item of the Ottawa Self-Injury Inventory (OSI) (Jeammet, 2003); ➂ Achieved a total score of ≥ 8 on the 24-item Hamilton Depression Rating Scale (HAMD-24) (Hamilton, 1967); ➃ Aged 12 to 35 years; ➄ Attained an educational level of primary school or higher; ➅ Demonstrated normal intelligence quotient (IQ). Exclusion Criteria: ➀ Past or current comorbidity with other psychiatric disorders, organic brain diseases, or severe somatic illnesses; ➁ History of substance dependence or substance abuse. The NSSI group was further stratified into severe and mild NSSI subgroups: participants with a score ≥ 3 on the past-year self-injury frequency item of the OSI were assigned to the severe NSSI subgroup, while those with a score < 3 were categorized into the mild NSSI subgroup.

Inclusion Criteria for the non-NSSI Group: ➀ Met the DSM-5 diagnostic criteria for depressive disorder but not for NSSI; ➁ Scored 0 on both the past-year and prior-year self-injury frequency items of the OSI, with no history of suicidal or self-harming behaviors; Criteria ➂ to ➅ were identical to those of the NSSI group. The exclusion criteria for the non-NSSI group were consistent with those applied to the NSSI group.

Inclusion Criteria for the HC Group: ➀ No lifetime or current diagnosis of any psychiatric disorder; ➁ HAMD-24 total score < 8; ➂ No organic brain lesions, severe somatic comorbidities, or history of substance dependence or abuse; Criteria ➃ to ➅ were identical to those of the NSSI group.

Ultimately, 68 participants were enrolled in the NSSI group, 40 in the non-NSSI group, and 32 in the HC group. The overall study sample was divided by age into an adolescent group (< 18 years) and an adult group (≥ 18 years). Both the adolescent group and the adult group were further subdivided into the NSSI group, the non-NSSI group, and the HC group. Within the NSSI group, participants were classified into the severe NSSI subgroup and the mild NSSI subgroup.

### 2.2 Assessment tools

#### 2.2.1 OSI

The OSI is a validated self-report tool designed for the assessment of NSSI behaviors. This scale contains dedicated items evaluating the frequency of NSSI, with the core question regarding past-year NSSI frequency phrased as: “You engaged in self-harming behaviors without suicidal intent; how many times did this happen in the past year?” The corresponding response options and scoring criteria are: never (0 points), 1–5 times (1 point), once per month (2 points), once per week (3 points), and daily (4 points). Higher scores reflect more frequent and severe NSSI behaviors. The Chinese version of the OSI has established favorable reliability and validity, with a test-retest reliability of 0.807 for the NSSI frequency subscale (Zhang et al., 2015).

#### 2.2.2 HAMD-24

As the most extensively used clinical instrument for assessing depressive symptom severity, the HAMD-24 boasts robust reliability and validity. The majority of items are rated on a 5-point ordinal scale ranging from 0 to 4. The total scale score correlates closely with depressive severity, wherein elevated scores correspond to more pronounced depressive symptomatology. Per the cutoff thresholds validated by Davis JM, a total score < 8 signifies no clinically significant depression, a score > 20 indicates mild-to-moderate depression, and a score > 35 denotes severe depression. The HAMD-24 comprises seven distinct factor dimensions: anxiety/somatization, weight loss, cognitive disturbance, diurnal variation, intellectual disability, sleep disturbance, and despair.

#### 2.2.3 BCIS

The BCIS is a validated self-report instrument developed by Beck et al. (2004) designed to assess cognitive insight among psychiatric populations. Comprising 15 items rated on a 0–3 Likert-type scale, this tool evaluates two core subdomains of cognitive insight: self-reflectiveness and self-certainty. Self-reflectiveness refers to the ability to recognize the inaccuracy of one’s own illness-related experiences and accept feedback from others, whereas self-certainty reflects absolute confidence in one’s current beliefs and belief systems. A composite index, computed as the self-reflectiveness score minus the self-certainty score, serves as a comprehensive indicator of global cognitive insight. Elevated scores on both self-reflectiveness and composite index correspond to superior cognitive insight (Ji et al., 2022). The Chinese (Taiwan) edition of the BCIS has been validated and exhibits favorable psychometric properties, including satisfactory reliability and validity (Kao and Liu, 2010).

### 2.3 Statistical analysis

All data collation, processing and statistical analyses were performed using IBM SPSS Statistics (Version 23.0). Chi-square tests, independent-samples *t*-tests, and one-way analysis of variance (ANOVA) were employed to compare intergroup differences in demographic characteristics and clinical data across all study cohorts. Pearson correlation analysis was conducted to explore the correlations between cognitive insight indicators and HAMD-24-related scores that showed significant discrepancies between the severe and mild NSSI subgroups. Subsequently, stepwise multiple linear regression analysis was performed to identify the independent influencing factors, with cognitive insight measures presenting significant intergroup differences as the dependent variables and statistically significant HAMD-24-related scores as the independent variables. A two-tailed significance level (α) of 0.05 was set for all statistical tests, and *P* < 0.05 was defined as statistically significant.

## 3 Results

### 3.1 Comparison of total sample among NSSI, non-NSSI, and healthy control groups

#### 3.1.1 Comparison of demographic characteristics, BCIS and HAMD-24

Table 1 shows that no significant intergroup differences were observed in age, gender distribution, years of education and BCIS scores across the NSSI group, non-NSSI group and HC group (all *P* > 0.05). Additionally, the NSSI group presented significantly higher total HAMD-24 score, cognitive disturbance and despair factors relative to the non-NSSI group (*P* < 0.05 or better).

**TABLE 1:** Comparison of demographic characteristics, BCIS and HAMD-24.

Variable	NSSI group (*n* = 68)	Non-NSSI group(*n* = 40)	HC group (*n* = 32)	F/χ 2/t	*P*
Age	18.78 ± 4.50	19.58 ± 4.21	19.91 ± 4.88	0.813	0.445
Gender(male/female)	18/50	12/28	13/19	2.062	0.357
Education(year)	10.06 ± 2.56	10.45 ± 3.11	10.31 ± 3.59	0.234	0.792
Self-certainty	9.07 ± 2.67	9.35 ± 3.13	8.84 ± 3.60	0.252	0.777
Self-reflectiveness	13.04 ± 3.15	14.33 ± 3.50	14.13 ± 3.81	2.167	0.118
Composite index	3.82 ± 3.74	5.18 ± 3.71	5.09 ± 4.73	1.915	0.151
Total HAMD-24 score	32.03 ± 5.85	27.50 ± 7.40	−	3.441	0.001**
Anxiety/somatization	7.29 ± 1.70	6.68 ± 2.46	−	1.503	0.136
Weight loss	0.50 ± 0.74	0.55 ± 0.68	−	−0.344	0.732
Cognitive disturbance	8.24 ± 2.63	6.63 ± 3.53	−	2.648	0.009**
Diurnal variation	0.92 ± 0.55	1.02 ± 0.83	−	−0.772	0.442
Intellectual disability	5.56 ± 1.03	5.03 ± 1.76	−	1.949	0.540
Sleep disturbance	3.71 ± 1.25	3.55 ± 1.48	−	0.586	0.559
Despair	5.53 ± 1.72	4.68 ± 2.38	−	2.108	0.038*

*P < 0.05,

**P ≤ 0.01.

#### 3.1.2 Associations among NSSI severity, BCIS and HAMD-24

##### 3.1.2.1 Comparison of demographic characteristics, BCIS and HAMD-24

Table 2 shows that no significant intergroup differences were detected in age, gender distribution or years of education between the severe NSSI subgroup (*n* = 38) and mild NSSI subgroup (*n* = 30) (all *P* > 0.05). The severe NSSI subgroup exhibited significantly lower self-reflectiveness and composite index compared with the mild NSSI subgroup (*P* < 0.05), with no significant between-group difference found in self-certainty (*P* > 0.05). Moreover, the severe NSSI subgroup showed markedly higher total HAMD-24 score, cognitive disturbance and despair factors than the mild NSSI subgroup (*P* < 0.05) or better, while no significant differences were observed in other HAMD-24 factors across the two subgroups (all *P* > 0.05).

**TABLE 2:** Comparison of demographic characteristics, BCIS and HAMD-24.

Variable	Mild NSSI group (*n* = 30)	Severe NSSI group(*n* = 38)	t/χ 2	*P*
Age	18.47 ± 3.88	19.03 ± 4.96	−0.507	0.614
Gender(male/female)	9/21	9/29	0.344	0.558
Education(year)	10.40 ± 2.77	9.79 ± 2.38	0.975	0.333
Self-certainty	8.90 ± 2.63	9.21 ± 2.72	−0.474	0.637
Self-reflectiveness	13.90 ± 2.52	12.37 ± 3.45	2.037	0.046*
Composite index	4.87 ± 3.77	3.00 ± 3.56	2.094	0.040*
Total HAMD-24 score	30.18 ± 6.29	33.56 ± 5.05	−2.381	0.022*
Anxiety/somatization	7.11 ± 1.85	7.44 ± 1.52	−0.674	0.439
Weight loss	0.46 ± 0.69	0.53 ± 0.79	−0.440	0.734
Cognitive disturbance	7.04 ± 2.44	9.24 ± 2.38	−3.656	0.001**
Diurnal variation	0.79 ± 0.57	1.03 ± 0.52	−2.059	0.084
Intellectual disability	5.39 ± 1.07	5.71 ± 1.00	−1.256	0.239
Sleep disturbance	3.82 ± 1.25	3.62 ± 1.26	0.496	0.526
Despair	4.93 ± 1.96	6.03 ± 1.34	−2.619	0.011*

*P < 0.05,

**P ≤ 0.01.

##### 3.1.2.2 Correlation analysis of BCIS and HAMD-24

Table 3 shows that within the severe NSSI subgroup, the composite index was significantly negatively correlated with the total HAMD-24 score (*r* = 0.366, *P* < 0.05), whereas its correlation with the cognitive disturbance factor was negative but not statistically significant (*P* > 0.05). The self-reflectiveness was negatively associated with the total HAMD-24 score and cognitive disturbance factor, yet these correlations did not reach statistical significance (all *P* > 0.05). Furthermore, neither the self-reflectiveness nor the composite index showed a significant correlation with the despair factor in the severe NSSI subgroup.

**TABLE 3:** Correlation analysis of self-reflectiveness, composite index and HAMD-24 in the severe NSSI subgroup.

HAMD-24	Self-reflectiveness	*P*	Composite index	*P*
Total HAMD-24 score	−0.063	0.725	−0.366	0.034*
Cognitive disturbance	−0.206	0.243	−0.251	0.152
Despair	0.186	0.292	−0.151	0.395

*P < 0.05.

##### 3.1.2.3 Stepwise multiple linear regression analysis

Stepwise multiple linear regression analysis demonstrated that the cognitive disturbance factor on the HAMD-24 was an independent determinant of self-reflectiveness in depressed patients with NSSI (β = −0.326, *P* < 0.05), with elevated cognitive disturbance factor corresponding to diminished self-reflective capacity. Furthermore, the total HAMD-24 score emerged as an independent predictor of the composite index in this cohort (β = −0.377, *P* < 0.05), and higher total HAMD-24 score were associated with lower composite index.

### 3.2 Age stratification analyses: adolescent and adult subgroups

#### 3.2.1 Adolescent subgroup

##### 3.2.1.1 Comparison of demographic characteristics, BCIS and HAMD-24

Table 4 shows that no significant intergroup differences were detected in age, gender distribution, years of education or BCIS among the NSSI subgroup (n = 31), non-NSSI subgroup (*n* = 18) and HC subgroup (*n* = 14) (all *P* > 0.05). The NSSI subgroup exhibited a significantly higher HAMD-24 anxiety/somatization factor than the non-NSSI subgroup (*P* < 0.05), while no significant between-group differences were found in the total HAMD-24 score and other HAMD-24 factors (all *P* > 0.05).

**TABLE 4:** Comparison of demographic characteristics, BCIS and HAMD-24 in adolescents.

Variable	NSSI group (*n* = 31)	Non-NSSI group(n = 18)	HC group (*n* = 14)	*F*/χ ^2^/*t*	*P*
Age	15.13 ± 1.38	15.78 ± 1.40	15.43 ± 1.56	1.188	0.312
Gender (male/female)	8/23	6/12	7/7	2.540	0.281
Education(year)	8.71 ± 2.05	9.61 ± 2.87	8.50 ± 3.16	0.942	0.396
Self-certainty	9.48 ± 2.85	10.11 ± 2.91	9.79 ± 3.47	0.250	0.779
Self-reflectiveness	13.23 ± 2.83	14.39 ± 3.27	14.29 ± 4.91	0.807	0.451
Composite index	3.65 ± 4.02	4.78 ± 3.92	3.79 ± 4.92	0.437	0.648
Total HAMD-24 score	30.96 ± 7.19	27.83 ± 8.58	−	1.336	0.188
Anxiety/somatization	7.36 ± 2.04	6.00 ± 2.22	−	2.126	0.039*
Weight loss	0.25 ± 0.52	0.56 ± 0.70	−	−1.693	0.097
Cognitive disturbance	8.21 ± 3.02	6.39 ± 3.73	−	1.824	0.075
Diurnal variation	0.86 ± 0.59	1.06 ± 0.87	−	−0.921	0.362
Intellectual disability	5.29 ± 1.08	5.39 ± 1.88	−	−0.236	0.814
Sleep disturbance	3.50 ± 1.43	3.56 ± 1.29	−	−0.134	0.894
Despair	5.54 ± 1.86	4.67 ± 2.22	−	1.434	0.159

*P < 0.05.

##### 3.2.1.2 Associations among NSSI severity, BCIS and HAMD-24

###### 3.2.1.2.1 Comparison of demographic characteristics, BCIS and HAMD-24

Table 5 shows that no significant intergroup differences were detected in age, gender distribution, years of education or self-certainty between the severe NSSI subgroup (*n* = 18) and mild NSSI subgroup (*n* = 13) (all *P* > 0.05). The severe NSSI subgroup exhibited significantly lower self-reflectiveness and composite index compared with the mild NSSI subgroup (*P* < 0.05 or better). Additionally, the severe NSSI subgroup presented markedly higher total HAMD-24 score, cognitive disturbance and diurnal variation factor relative to the mild NSSI subgroup (*P* < 0.05 or better), with no significant between-group differences observed in other HAMD-24 factor scores (all *P* > 0.05).

**TABLE 5:** Comparison of demographic characteristics, BCIS and HAMD-24 in adolescents.

Variable	Mild NSSI group (*n* = 13)	Severe NSSI group(*n* = 18)	t/χ ^2^	*P*
Age	15.31 ± 1.25	15.00 ± 1.50	0.604	0.550
Gender(male/female)	2/11	6/12	1.270	0.260
Education(year)	8.54 ± 2.30	8.83 ± 1.92	−0.389	0.700
Self-certainty	9.38 ± 2.73	9.56 ± 3.01	−0.165	0.870
Self-reflectiveness	15.08 ± 2.67	11.89 ± 2.14	3.698	0.001**
Composite index	5.54 ± 3.78	2.28 ± 3.71	2.390	0.024*
Total HAMD-24 score	27.92 ± 7.51	33.25 ± 6.22	−2.054	0.050*
Anxiety/somatization	6.92 ± 2.50	7.69 ± 1.62	−0.930	0.365
Weight loss	0.17 ± 0.39	0.31 ± 0.60	−0.731	0.472
Cognitive disturbance	6.58 ± 2.43	9.44 ± 2.90	−2.831	0.009**
Diurnal variation	0.58 ± 5.15	1.06 ± 0.57	−2.283	0.031*
Intellectual disability	5.08 ± 1.17	5.44 ± 1.03	−0.851	0.402
Sleep disturbance	3.58 ± 1.51	3.44 ± 1.41	0.260	0.797
Despair	5.00 ± 2.13	5.94 ± 1.57	−1.343	0.191

*P < 0.05,

**P ≤ 0.01.

###### 3.2.1.2.2 Correlation analysis of BCIS and HAMD-24

Table 6 shows that in the severe NSSI subgroup, both self-reflectiveness and composite index were significantly negatively correlated with the total HAMD-24 score (*r* = −0.514, *r* = −0.559, all *P* < 0.05) and diurnal variation factor (*r* = −0.505, *r* = −0.538, *P* < 0.05), whereas their correlations with the cognitive disturbance factor were negative but not statistically significant (*P* > 0.05).

**TABLE 6:** Correlation analysis of self-reflectiveness, composite index and HAMD-24 in the severe NSSI subgroup.

HAMD-24	Self-reflectiveness	*P*	Composite index	*P*
Total HAMD-24 score	−0.514	0.042*	−0.559	0.024*
Cognitive disturbance	−0.476	0.062	−0.180	0.505
Diurnal variation	−0.505	0.046*	−0.538	0.031*

*P < 0.05.

###### 3.2.1.2.3 Stepwise multiple linear regression analysis

Stepwise multiple linear regression analysis demonstrated that the total HAMD-24 score was an independent determinant of self-reflectiveness among depressed adolescents with NSSI (β = −0.734, *P* < 0.05), with higher total HAMD-24 score corresponding to diminished self-reflective capacity. Moreover, the HAMD-24 diurnal variation factor emerged as an independent predictor of composite index in this adolescent subgroup (β = −0.583, *P* < 0.05), and elevated diurnal variation factor were associated with lower composite index.

#### 3.2.2 Adult subgroup

##### 3.2.2.1 Comparison of demographic characteristics, BCIS and HAMD-24

Table 7 shows that no significant intergroup differences were detected in age, gender distribution, years of education or BCIS among the NSSI subgroup (*n* = 37), non-NSSI subgroup (*n* = 22) and HC subgroup (*n* = 18) (all *P* > 0.05). The NSSI subgroup exhibited significantly higher total HAMD-24 score and intellectual disability factor compared with the non-NSSI subgroup (*P* ≤ 0.01), with no significant between-group differences observed in other HAMD-24 factors (all *P* > 0.05).

**TABLE 7:** Comparison of demographic characteristics, BCIS and HAMD-24 in adults.

Variable	NSSI group (*n* = 37)	Non-NSSI group(*n* = 22)	HC group (*n* = 18)	*F*/χ ^2^/*t*	*P*
Age	21.84 ± 3.86	22.68 ± 2.98	23.39 ± 3.48	1.230	0.298
Gender(male/female)	10/27	6/16	6/12	0.261	0.877
Education(year)	11.19 ± 2.41	11.14 ± 3.18	11.72 ± 3.32	0.258	0.773
Self-certainty	8.73 ± 2.49	8.73 ± 3.24	8.11 ± 3.63	0.292	0.748
Self-reflectiveness	12.89 ± 3.43	14.27 ± 3.76	14.00 ± 2.83	1.347	0.266
Composite index	3.97 ± 3.54	5.50 ± 3.58	6.11 ± 4.46	2.312	0.106
Total HAMD-24 score	32.91 ± 4.37	27.23 ± 6.47	−	3.931	0.000**
Anxiety/somatization	7.24 ± 1.33	7.23 ± 2.56	−	0.000	0.989
Weight loss	0.71 ± 0.84	0.55 ± 0.67	−	0.756	0.453
Cognitive disturbance	8.26 ± 2.30	6.82 ± 3.43	−	1.891	0.064
Diurnal variation	0.97 ± 0.52	1.00 ± 0.82	−	−0.165	0.870
Intellectual disability	5.79 ± 0.95	4.73 ± 1.64	−	7.672	0.010**
Sleep disturbance	3.88 ± 1.07	3.55 ± 1.65	−	0.928	0.357
Despair	5.53 ± 1.64	4.68 ± 2.55	−	1.916	0.176

**P ≤ 0.01.

##### 3.2.2.2 Comparisons of demographic characteristics, BCIS and HAMD-24 between severe and mild NSSI subgroups

Table 8 shows that no significant intergroup differences were observed in age, gender distribution, years of education or BCIS between the severe NSSI subgroup (*n* = 20) and mild NSSI subgroup (*n* = 17) (all *P* > 0.05). The severe NSSI subgroup presented markedly higher HAMD-24 cognitive disturbance and despair factors relative to the mild NSSI subgroup (*P* < 0.05), whereas no significant differences were detected in the total HAMD-24 score and other HAMD-24 factors across the two subgroups (all *P* > 0.05).

**TABLE 8:** Comparison of demographic characteristics, BCIS and HAMD-24 in adults.

Variable	Mild NSSI group (*n* = 17)	Severe NSSI group(*n* = 20)	*t*/χ ^2^	*P*
Age	20.88 ± 3.44	22.65 ± 4.10	−1.406	0.169
Gender(male/female)	9/21	9/29	0.344	0.558
Education(year)	11.82 ± 2.24	10.65 ± 2.48	1.499	0.143
Self-certainty	8.53 ± 2.58	8.90 ± 2.47	−0.446	0.658
Self-reflectiveness	13.00 ± 2.06	12.80 ± 4.32	0.174	0.863
Composite index	4.35 ± 3.79	3.65 ± 3.38	0.597	0.555
Total HAMD-24 score	31.88 ± 4.76	33.83 ± 3.90	−1.303	0.203
Anxiety/Somatization	7.25 ± 1.24	7.22 ± 1.44	0.061	0.952
Weight loss	0.69 ± 0.79	0.72 ± 0.90	−0.119	0.906
Cognitive disturbance	7.38 ± 2.47	9.06 ± 1.86	−2.254	0.031*
Diurnal variation	0.94 ± 0.57	1.00 ± 0.49	−0.344	0.733
Intellectual disability	5.63 ± 0.96	5.94 ± 0.94	−0.982	0.334
Sleep disturbance	4.00 ± 1.03	3.78 ± 1.11	0.601	0.552
Despair	4.88 ± 1.89	6.11 ± 1.13	−2.342	0.026*

*P < 0.05.

## 4 Discussion

Analysis of the overall study sample revealed no statistically significant differences in BCIS among the NSSI group, non-NSSI group, and HC group. Consistent results were also observed in both adolescent and adult subgroups following age stratification, indicating that the mere occurrence of NSSI is not a direct indicator of impaired cognitive insight. Nevertheless, further subgroup analysis demonstrated that the severe NSSI subgroup exhibited significantly lower BCIS self-reflectiveness and composite index relative to the mild NSSI subgroup in the overall cohort, suggesting that escalating NSSI severity is associated diminished cognitive insight, as reflected by reduced self-reflective capacity, in patients with depression. Accumulating evidence indicates that individuals engaging in NSSI represent a heterogeneous rather than homogeneous population (He et al., 2023). Prior studies have stratified subjects into NSSI subgroups of varying severity based on two core features: the form of NSSI and the total count of distinct NSSI behaviors, and identified divergent patterns of cognitive dysfunction across these subgroups. Specifically, the mild NSSI subgroup was characterized by impaired inhibitory control, while the severe NSSI subgroup presented with prominent working memory deficits (Fikke et al., 2011). Cognitive insight constitutes a form of metacognition, representing a higher-order cognitive processing modality (Kao and Liu, 2010). Although multifactorial etiologies contribute to metacognitive impairment, executive dysfunction represents a key underlying mechanism. As a core component of executive function (Chen M. et al., 2023), working memory refers to the ability to temporarily store and manipulate information, and serves as the fundamental basis for executing complex cognitive tasks. From a neurobiological perspective, metacognition and working memory share overlapping functional brain regions, with the prefrontal cortex (PFC) serving as a critical shared substrate (Fleming and Dolan, 2012; Maniscalco and Lau, 2015). Self-reflectiveness, a core dimension of cognitive insight, inherently engages verbal working memory and decision-making processes (Orfei et al., 2013). Existing research has established significant associations between self-reflectiveness and neural activation in the bilateral ventromedial prefrontal cortex (VMPFC) (van der Meer et al., 2013) and bilateral ventrolateral prefrontal cortex (VLPFC) (Buchy et al., 2015). Further magnetic resonance imaging (MRI) studies exploring whole-brain correlates of cognitive insight have demonstrated a positive correlation between self-reflectiveness scores and gray matter volume in the right VLPFC. Given that the VLPFC mediates the generation and maintenance of self-referential hypotheses within working memory, VLPFC atrophy is postulated to underlie the reduced dialectical reasoning about abnormal experiences in affected patients, thereby compromising cognitive insight (Orfei et al., 2013). In conjunction with these prior findings, the present study further demonstrates that the severity of cognitive insight impairment in depressed patients with NSSI is closely linked to NSSI severity. The observed cognitive insight deficits in the severe NSSI subgroup may be partially attributable to underlying working memory impairments, a hypothesis that warrants rigorous validation in future mechanistic investigations.

Depressive symptoms are tightly linked to NSSI, and a growing body of evidence has validated that depressive symptoms serve as a critical predictor of NSSI among adolescents (Kang et al., 2021). Previous research has also established a significant positive correlation between depressive disorders and adolescent NSSI (Tang et al., 2022). Prior studies have documented that depressed patients with NSSI present markedly higher total HAMD-24 score than those without NSSI, indicative of more severe depressive symptomatology in the former group (Zhang et al., 2022). Consistent with these established findings, our overall sample analysis revealed elevated total HAMD-24 score in depressed patients with NSSI relative to those without NSSI. Furthermore, our results extend prior observations by indicating that this patient population not only exhibits more severe depression, but also manifests more pronounced cognitive distortions and chronic despair.

Subgroup analysis of the total sample stratified by NSSI severity in the present study revealed that the severe NSSI subgroup exhibited significantly higher total HAMD-24 score, cognitive disturbance and despair factors than the mild NSSI subgroup. Correlation analysis verified a significant negative correlation between composite index and total HAMD-24 score within the severe NSSI subgroup. Moreover, stepwise linear regression analysis further determined that the HAMD-24 cognitive disturbance factor serves as an independent determinant of self-reflective capacity, while total HAMD-24 score acts as an independent predictor of composite index. Collectively, these findings imply an inherent association between NSSI severity and cognitive insight from the perspective of the interplay between clinical symptoms and cognitive function. Patients with severe NSSI commonly present with more severe depressive symptoms, more prominent cognitive distortions and chronic despair, and the underlying mechanism can be elucidated via a bidirectional interactive pathway. On one hand, the sustained accumulation of cognitive impairment, despair and other negative symptoms in severe depression directly diminishes individuals’ self-awareness, self-evaluation and self-reflection abilities, resulting in inadequate recognition of the hazards posed by their psychopathological status and NSSI behaviors, and thus further escalating the severity of self-injurious acts. On the other hand, impaired cognitive insight further compromises patients’ capacity to perceive and manage depressive symptoms, hindering their ability to actively evade negative emotions and rectify cognitive biases, which subsequently perpetuates the exacerbation of depressive symptomatology. This accounts for the significant negative correlation between cognitive insight metrics and HAMD-24 scores detected in the present study.

Upon age stratification in the present study, further subgroup analyses revealed that the significantly lower self-reflectiveness and composite index in the severe NSSI subgroup versus the mild NSSI subgroup were solely evident in the adolescent cohort, with no statistically significant intergroup differences in cognitive insight parameters detected in the adult subgroup. These findings indicate that the association between NSSI severity and cognitive insight among depressed patients may be age-dependent. Adolescence constitutes a critical developmental window for PFC maturation. During the transition from adolescence to adulthood, individuals’ higher-order cognitive functions undergo continuous refinement, paralleled by progressive structural and functional maturation of the brain regions mediating these cognitive processes (Larsen and Luna, 2018). Neuroimaging evidence confirms that association cortices, including the PFC, undergo sustained structural and functional remodeling throughout adolescence, encompassing global brain morphology, neural connectivity, and task-evoked activation profiles (Giedd, 2004; Gogtay et al., 2004; Luna et al., 2010; Luna et al., 2015; Marek et al., 2015; Simmonds et al., 2014; Sowell et al., 2004). The PFC serves as a pivotal brain region governing metacognition (Fleming and Dolan, 2012), emotional regulation (Dixon et al., 2017), and response inhibition (Rae et al., 2015), yet this region remains incompletely developed in adolescents. Extensive research has established a strong association between adolescent depression and executive dysfunction (Diaz et al., 2022). Adolescents are particularly prone to executive impairments when severe NSSI behaviors coincide with depressive symptomatology. Compared with depressed adolescents without NSSI, those exhibiting NSSI display significantly poorer cognitive flexibility, which further declines with increasing NSSI frequency (Wang et al., 2023), thus accounting for the more severe cognitive insight deficits observed in the severe NSSI adolescent subgroup in this study. Prior research has also documented that adolescents with major depressive disorder and severe NSSI perform significantly worse on spatial working memory (SWM) tasks than their counterparts with mild NSSI (Fikke et al., 2011). In contrast, adult patients present with more fully developed PFC relative to adolescents. Additionally, adult patients exhibit high heterogeneity in disease duration (Weavers et al., 2026) and substantial variability in treatment histories (Hunter et al., 2012), with some individuals demonstrating partial improvements in executive function following standardized therapeutic interventions (Groves et al., 2021), which may mitigate the detrimental effects of NSSI severity on cognitive insight. Collectively, these multifaceted factors likely underlie the lack of significant intergroup differences in cognitive insight measures between severe and mild NSSI subgroups in the adult cohort.

The present study revealed significantly elevated anxiety/somatization factor among adolescents with NSSI in the adolescent subgroup. Prior research has established a significant correlation between anxiety and NSSI onset, with anxious symptomatology identified as a potential risk factor for NSSI (Shi et al., 2025). Stratified analyses within the adolescent cohort showed that the severe NSSI subgroup exhibited markedly higher total HAMD-24 score, cognitive disturbance factor, and diurnal variation factor relative to the mild NSSI subgroup. Furthermore, self-reflectiveness and composite index were significantly negatively correlated with total HAMD-24 score and diurnal variation factor in this subgroup. Subsequent stepwise regression analysis demonstrated that total HAMD-24 score independently predicted self-reflective capacity, while diurnal variation factor independently predicted composite index levels. Diurnal mood fluctuations may reflect the severity risk of depression (Courtet and Olié, 2012). Compared with previous data from adult patients, diurnal mood alterations are more prevalent in adolescent patients with severe depression and comorbid anxiety symptoms, and those with such fluctuations present with more severe depressive symptomatology, primarily manifesting as more severe cognitive dysfunction, exacerbated somatic distress, and heightened despair (Duan et al., 2025). The current findings indicate that diurnal mood changes may be closely associated with impaired cognitive insight in adolescent depression patients with severe NSSI.

The present study revealed that within the adult subgroup, only the total HAMD-24 score and intellectual disability factor were significantly elevated in the NSSI group relative to the non-NSSI group. Among adult participants, the severe NSSI subgroup merely showed higher cognitive disturbance and despair factors compared with the mild NSSI subgroup, with no statistically significant difference in HAMD-24 total score detected between the two subgroups. The intellectual disability factor encompasses items assessing psychomotor intellectual disability. With respect to this symptom, existing literature indicates that depressed patients may manifest both motor and cognitive intellectual disability (van Hoof et al., 1998), whereas other studies have reported that psychomotor intellectual disability in unipolar depression patients involves only motor deficits without concomitant cognitive impairment (Bange and Bathien, 1998). When confronted with negative affective states, individuals may engage in NSSI as a form of self-punishment, seeking to replace feelings of despair with a spurious sense of self-control to mitigate emotional distress and achieve transient relief and relaxation (Kang et al., 2021). Accordingly, despair may drive individuals to adopt NSSI as an “emotional escape route.” Of note, some adult patients suffer from fluctuating disease courses, persistent clinical symptoms, or dissatisfaction with treatment efficacy; these adverse conditions can further induce despair and ultimately elevate the risk of NSSI (Wang et al., 2021).

The novelty of this study resides in focusing on patients with depression comorbid NSSI, systematically characterizing their cognitive insight profiles, and further investigating the associations between BCIS and HAMD-24. The findings revealed that relative to patients with mild NSSI, those with severe NSSI exhibited significantly poorer self-reflection capacity and lower composite index, indicative of overt cognitive insight impairment in this subgroup, accompanied by markedly compromised ability to evaluate and rectify distorted beliefs and cognitive biases. Furthermore, depressive symptom severity was positively correlated with the degree of cognitive insight deficit, suggesting that ameliorating depressive symptoms in patients with severe NSSI may enhance their objective appraisal and accurate recognition of abnormal mental states, strengthen the capacity to correct misconceptions, and consequently reduce the likelihood of NSSI. These results carry substantial clinical implications for diagnostic and therapeutic practice. For the assessment and intervention of cognitive insight in depressed patients with NSSI, clinicians should not only refine the stratification of NSSI severity but also fully account for age-related differences, discarding the simplistic binary criterion of “NSSI presence versus absence.” Instead, differentiated targeted strategies are warranted for adolescent and adult patient populations. For adolescents, we recommend prioritizing NSSI severity, cognitive insight, total HAMD-24 score, diurnal mood fluctuation, and anxiety/somatization symptoms as core evaluation indices. With reducing overall depressive severity as the primary objective, tailored interventions including metacognitive training, self-reflection facilitation, and cognitive behavioral therapy (CBT) should be delivered, combined with emotion regulation and anxiety alleviation strategies, to interrupt the vicious cycle between depressive symptoms and self-injurious acts and lower the risk of severe NSSI. For adult patients, the core clinical focus is mitigating overall depressive burden, with targeted efforts on improving cognitive dysfunction, relieving despair, and alleviating psychomotor intellectual disability. Comprehensive interventions should be implemented in conjunction with assessments of patients’ real-life stressors and social support resources, and personalized regimens should be formulated to address chronic depressive distress, thereby reducing the incidence of NSSI. Collectively, this study highlights that systematic evaluation and targeted intervention of cognitive insight are imperative when designing individualized treatment plans, as this approach not only improves treatment adherence and optimizes clinical outcomes but also furnishes a theoretical foundation for developing evidence-based psychosocial intervention strategies.

As a preliminary exploratory study, the present investigation is not without limitations. First, prior evidence suggests a potential interplay between cognitive insight and executive function. Future studies should integrate comprehensive neurocognitive assessment batteries to further elucidate the underlying mechanistic associations between these constructs. Second, given its cross-sectional design, this study can only establish correlational relationships among NSSI, depressive symptoms, and cognitive insight, but it cannot infer causal pathways or dynamic temporal trajectories. Consequently, longitudinal cohort studies are warranted to validate these findings. Third, the severity of NSSI in the current work was defined solely by incident frequency; however, NSSI morphology and the degree of physical injury are also critical determinants. Thus, subsequent research should implement a more holistic, multi-dimensional framework to evaluate NSSI severity. Fourth, a subset of participants was prescribed antidepressant medications at enrollment. While efforts were made to control for this factor, the potential confounding effects of pharmacotherapy on cognitive function cannot be completely excluded. Finally, the study is limited by its relatively underpowered sample size. Conclusions drawn herein require further validation through larger-scale, multi-center investigations to enhance the generalizability of our findings.

In conclusion, the present study demonstrated that patients with depression comorbid NSSI, especially the severe NSSI subgroup, present cognitive insight impairment featured by declined self-reflection ability and lower composite index. Such impairment is closely correlated with depressive symptom severity, and global depressive burden as well as cognitive dysfunction are the major influencing factors for cognitive insight deficit in these patients. Further analysis uncovered an age-dependent association: cognitive insight impairment is significantly linked to depressive symptoms in adolescent patients with severe NSSI depression, while no obvious evidence of such impairment is found in adult patients. These findings indicate that optimizing depression treatment strategies targeting cognitive dysfunction and diurnal mood variation (morning worsening, evening improvement) can improve cognitive insight in adolescent patients, enhance their ability to assess abnormal mental states and correct misconceptions, thus reducing the risk of NSSI.

## Data Availability

The raw data supporting the conclusions of this article will be made available by the authors, without undue reservation.
